# Novel Humanized Peripheral Blood Mononuclear Cell Mouse Model with Delayed Onset of Graft-versus-Host Disease for Preclinical HIV Research

**DOI:** 10.1128/JVI.01394-21

**Published:** 2022-02-09

**Authors:** Leo Holguin, Liliana Echavarria, John C. Burnett

**Affiliations:** a Center for Gene Therapy, Beckman Research Institute of City of Hope, Duarte, California, USA; b Irell & Manella Graduate School of Biological Sciences, City of Hope, Duarte, California, USA; c Center for Comparative Medicine, City of Hope, Duarte, California, USA; Emory University

**Keywords:** HIV, human immunodeficiency virus, humanized mouse model, antiretroviral therapy, antiviral agents, hu-PBMC, TKO, GVHD, graft-versus-host disease

## Abstract

Humanized mouse models are based on the engraftment of human cells in immunodeficient mouse strains, most notably the NSG strain. Most used models have a major limitation in common, the development of graft-versus-host disease (GVHD). GVHD not only introduces variabilities into the research data but also leads to animal welfare concerns. A new mouse strain, B6.129S-Rag2^tm1Fwa^ CD47^tm1Fpl^ Il2rg^tm1Wjl^/J, which lacks Rag1, IL2rg, and CD47 (triple knockout [TKO]), is resistant to GVHD development. We transplanted TKO mice with human peripheral blood mononuclear cells (PBMCs) to establish a new humanized PBMC (hu-PBMC) mouse model. A cohort of these mice was infected with HIV-1 and monitored for plasma HIV viremia and CD4^+^ T cell depletion. The onset and progression of GVHD were monitored by clinical signs. This study demonstrates that TKO mice transplanted with human PBMCs support engraftment of human immune cells in primary and secondary lymphoid tissues, rectum, and brain. Moreover, the TKO hu-PBMC model supports HIV-1 infection via the intraperitoneal, rectal, or vaginal route, as confirmed by robust plasma HIV viremia and CD4^+^ T cell depletion. Lastly, TKO mice showed a delayed onset of GVHD clinical signs (∼24 days) and exhibited significant decreases in plasma levels of tumor necrosis factor beta (TNF-β). Based on these results, the TKO hu-PBMC mouse model not only supports humanization and HIV-1 infection but also has a delayed onset of GVHD development, making this model a valuable tool in HIV research.

**IMPORTANCE** Currently, there is no cure or vaccine for HIV infection; thus, continued research is needed to end the HIV pandemic. While many animal models are used in HIV research, none is used more than the humanized mouse model. A major limitation with current humanized mouse models is the development of graft-versus-host disease (GVHD). Here, we describe a novel humanized-PBMC mouse model that has a delayed onset GVHD development and supports and models HIV infection comparably to well-established humanized mouse models.

## INTRODUCTION

Human immunodeficiency virus (HIV) is a global pandemic that currently affects 37 million people, with about 2.3 million new cases every year (1). Currently, there is no effective cure or prophylactic vaccine for HIV infection; therefore, continued research is needed to end the HIV pandemic. Animal models play an important role in finding new cures and vaccines. The use of humanized mouse models has gained popularity due to their “humanized” immune system (2–4), and these models have proven useful for the study of HIV replication, pathogenesis, transmission, and prevention. The most common humanized mouse models for HIV research are engrafted with human peripheral mononuclear blood cells (hu-PBMC), CD34^+^ hematopoietic stem cells (hu-CD34^+^), or a combination of CD34^+^ cells and the implantation of fetal liver and thymic tissue (hu-BLT) (5). All of these models involve the use of immunodeficient mouse strains—NSG (NOD.Cg-Prkdc^scid^ Il2rg^tm1Wjl^/Sz), NRG [NOD.129S7(B6)-Rag1^tm1Mom^ IL2rg^tm1Wjll^/Sz], NOG (NOD.Cg-Prkdc^scid^ Il2rg^tm1Sug^), and BRG [C.129(Cg)Rag2^tm1Fwa^ IL2rg^tm1Sug^/Jic]—which can support efficient, stable, and systemic engraftment of human cells and tissues (6).

The hu-PBMC model is a fast and logistically simple method for mouse humanization (5). Kim et al. described a simple method of transplanting human PBMC in nonirradiated NSG mice (7). In the NSG hu-PBMC model, CD45^+^, CD3^+^, CD4^+^, and CD8^+^ T cells were detected in peripheral blood, lymph nodes, spleen, and liver (7). The model supported HIV-1 infection with depletion of CD4^+^ T cell counts and high plasma viremia and responded to antiretroviral therapy (ART) with no detectable viremia or CD4^+^ T cell depletion (7). The hu-PBMC NSG model serves as a relevant HIV-1 infection and pathogenesis model that can be used in pharmacokinetic and pharmacodynamic (PK/PD) analysis of new therapies. The hu-PBMC model is particularly useful in preclinical studies that require autologous transplantation of T cells in HIV/AIDS patients after *ex vivo* manipulation, such as transduction with therapeutic vectors (8), generation of chimeric antigen receptor (CAR) T cells (9–11), and treatment with site-specific genome editing nucleases (12, 13).

Despite the broad utility of the hu-PBMC model, a major limitation is its high susceptibility to xenogeneic graft-versus-host disease (GVHD). In the hu-PBMC model, the onset of GVHD varies between 4 and 8 weeks after the transplantation of human PBMC (14). GVHD occurs when the engrafted human cells start to attack the mouse tissues, leading to the early euthanasia of mice and loss of valuable data (15). Clinically, GVHD presents as weight loss, hunching posture, hair loss, and decreased activity/lethargy (16). Besides limiting the experimental time of the animals, these clinical signs represent animal welfare concerns often in conflict with institutional animal care and use committee (IACUC) standards. Pathologically, GVHD is characterized by lymphocytic infiltration and sclerosis of the skin, gastrointestinal (GI) tract, and skin culminating in the early death of mice. Besides the loss of animals, GVHD can complicate experimental results by introducing GVHD-associated fibroinflammatory lesions (17). A potential solution is the development of a GVHD-resistant hu-BLT model. Lavender et al. developed a hu-BLT model that develops B and T cell immunity to HIV infection and is resistant to GVHD, using the mouse strain B6.129S-*Rag2^tm1Fwa^ CD47^tm1Fpl^ Il2rg^tm1Wjl^*/J (triple knockout [TKO]) (18). They observed that TKO hu-BLT mice did not develop any GVHD-related clinical signs over the 29 weeks of the experiment and in a subsequent study observed no GVHD development up to 45 weeks (19). TKO mouse knockout of *CD47* in addition to *Rag 1* and *IL2rg* allowed for reconstitution of human immune cells with little GVHD development (18, 19).

In this study, we hypothesized that TKO mice can be used to develop a novel hu-PBMC model that has a delayed onset of GVHD and supports HIV-1 infection. Here, we show that the TKO hu-PBMC model supports engraftment of human immune cells, supports HIV-1 infection, and showed a delayed onset of GVHD clinical signs (∼28 days) compared to NSG mice. Based on these results, the TKO hu-PBMC mouse model not only supports humanization and HIV-1 infection but also has a delayed onset of GVHD, making this model a valuable tool in HIV research.

## RESULTS

### TKO mice support the engraftment of human peripheral blood mononuclear cells.

**FIG 1:**
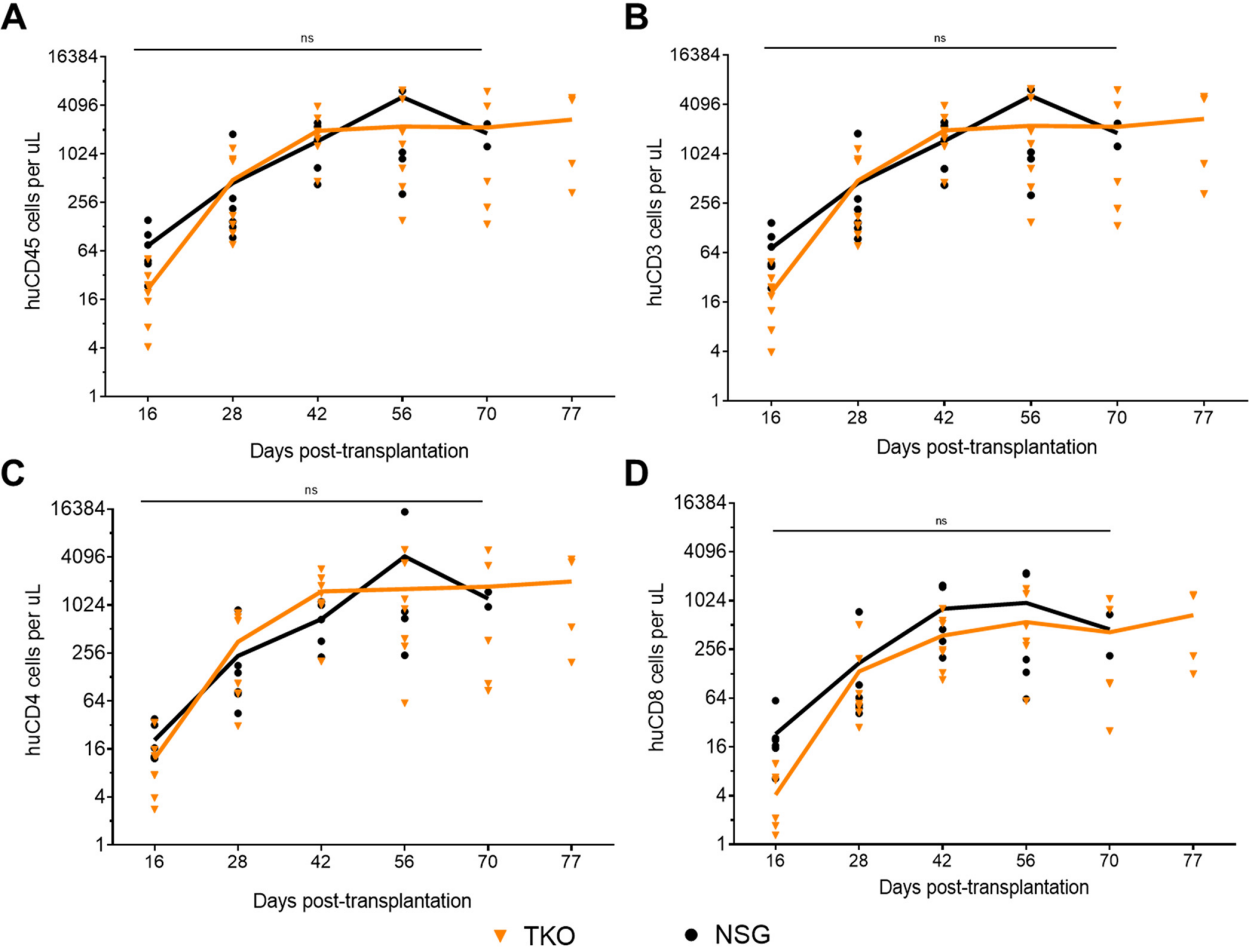
TKO mice support engraftment of human PBMC comparably to NSG mice. Four- to 6-week-old TKO (*n* = 7) and NSG (*n* = 6) mice were transplanted with 1.0 × 10^7^ human PBMC. Engraftment of human cells was checked by FACS analysis every 1 to 2 weeks for 11 weeks as shown in the graphs. Absolute cell numbers were calculated by using BD liquid counting beads. Similar to the NSG hu-PBMC mice, TKO mice support engraftment of CD45^+^ leukocytes (A), mainly CD3^+^ T cells (B), including robust numbers of CD4^+^ (C) and CD8^+^ T cells (D). (Three TKO mice were lost before experiment endpoint at days 70 and 77, while all 6 NSG mice were lost before experiment endpoint at days 42, 70, and 77.) Statistical significance was determined by unpaired *t* test using the Holm-Sidak method on Prism, with alpha value of 0.05.

To test whether TKO mice were able to support engraftment of human PBMC, we engrafted 4- to 6-week-old TKO mice with 10 million hu-PBMC by intraperitoneal (i.p.) injection as previously described (7). To compare the TKO mice with a standard PBMC model, we engrafted in parallel 4- to 6-week-old NSG mice with the same donor hu-PBMC and analyzed peripheral blood every 2 weeks after transplantation. TKO mice showed detectable and stable circulating human CD45^+^ leukocytes starting at 2 weeks after transplantation comparable to the NSG mice (Fig. 1A). The majority of the CD45^+^ cells in the peripheral blood were CD3^+^ lymphocytes, which was also seen in the NSG hu-PBMC model (Fig. 1B). Further analysis of the CD3^+^ cells showed that TKO mice support robust circulating CD4^+^ T cells (Fig. 1C) and CD8^+^ T cells (Fig. 1D), similar to the NSG mice. In TKO mice, CD4^+^ T cells peaked at around week 6 and remained relatively stable over the 11-week study. In comparison, NSG mice reached peak CD4^+^ T cells around week 8 (day 56), having a delay on peak circulating cells (Fig. 1C). This shows that the TKO hu-PBMC model can reliably engraft hu-PBMC and repopulate CD3^+^ lymphocytes, including HIV target cells: CD4^+^ T cells.

**FIG 2:**
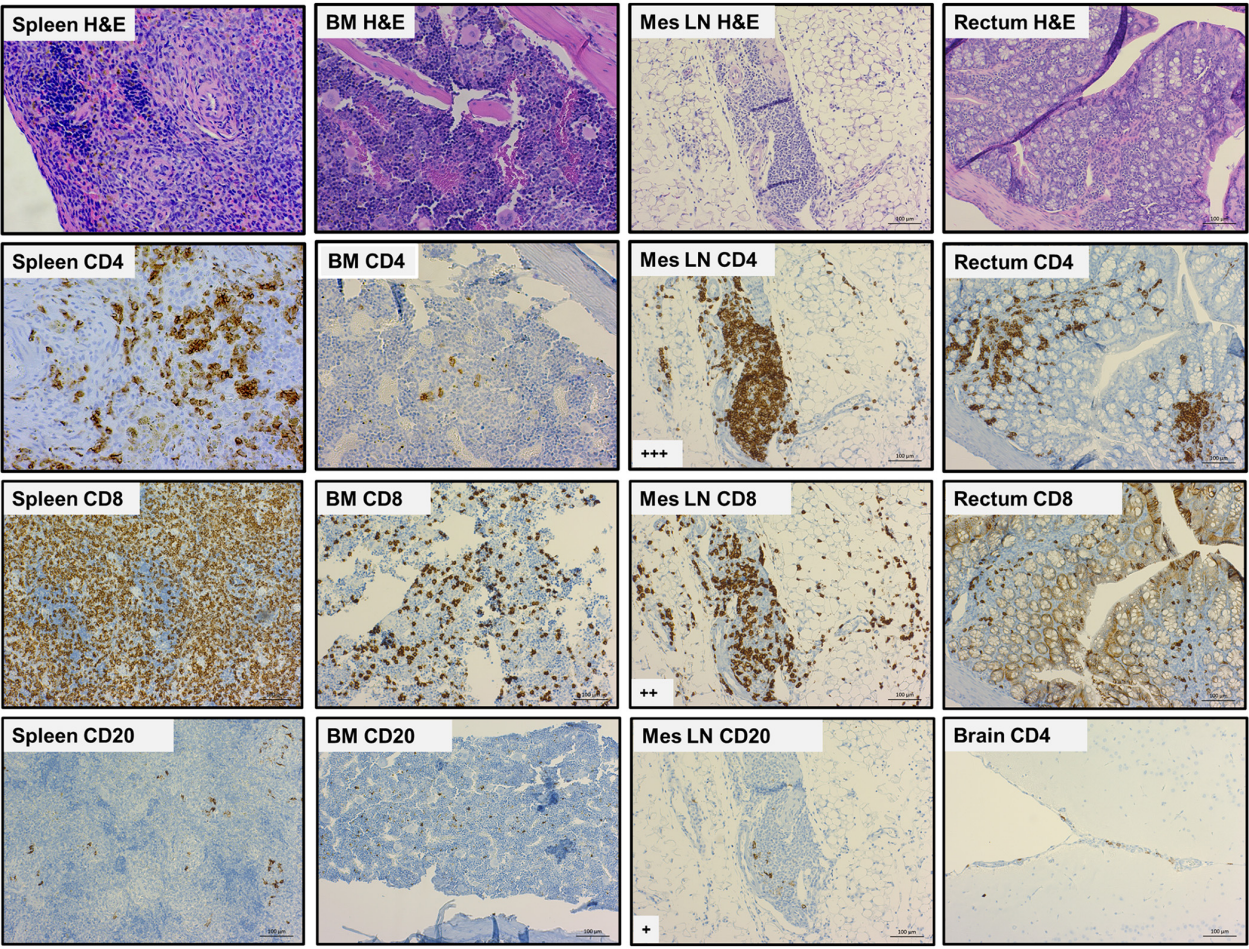
TKO hu-PBMC model supports engraftment of human immune cells in lymphoid tissues. TKO mice with robust and consistent peripheral CD45^+^ engraftment were humanely euthanized, and tissues were collected for histology and IHC. Tissues were processed by the COH Solid Tumor Pathology Core for histology and immunohistochemistry: H&E, huCD3^+^, huCD4^+^, huCD8^+^, huCD20^+^, and huCD163^+^. There was positive staining of CD3, CD4, CD8, CD20, and CD163 cells, indicating that the TKO hu-PBMC model supports engraftment of immune cells in lymphoid tissues. (Images for CD3, CD163, and GFAP are not shown). Representative images from the cohort of mice are shown. Staining scores are as defined for Table 1.

Next, we analyzed the development of lymphoid tissues in the mice. Lymphoid tissues are sites where abundant lymphocytes live and sites of antigen presentation and lymphocyte activation in humans, which is important for HIV-1 replication and maintenance of persistent HIV-1 infection (20). After the 11-week study, mice were humanely euthanized, and tissues were collected for immunohistochemistry analysis. TKO mice showed robust staining of CD3^+^ lymphocytes and CD4^+^ and CD8^+^ T cells in many tissues, including spleen, mesenteric lymph node, bone marrow, lung, liver, colon, rectum, and brain, while CD14^+^ CD163^+^ macrophages and CD20^+^ B cells were mainly seen in spleen and lymph node (Fig. 2 and Table 1). Collectively, the circulating CD4^+^ T cells (Fig. 1) and development of CD4^+^ T cell-rich lymphoid tissues (Fig. 2) suggest that the TKO hu-PBMC model may facilitate *in vivo* studies of HIV-1 infection.

**TABLE 1:**
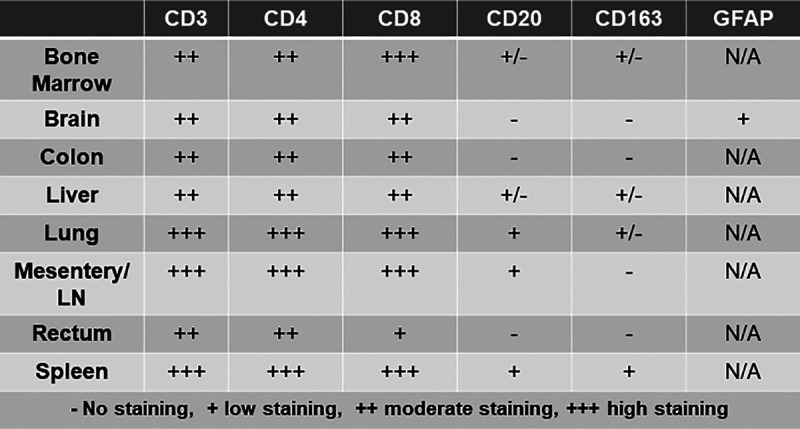
Chart of engraftment of human immune cells in different tissues^*a*^

a N/A, not applicable.

### TKO hu-PBMC model supports and models HIV-1 infection.

**FIG 3:**
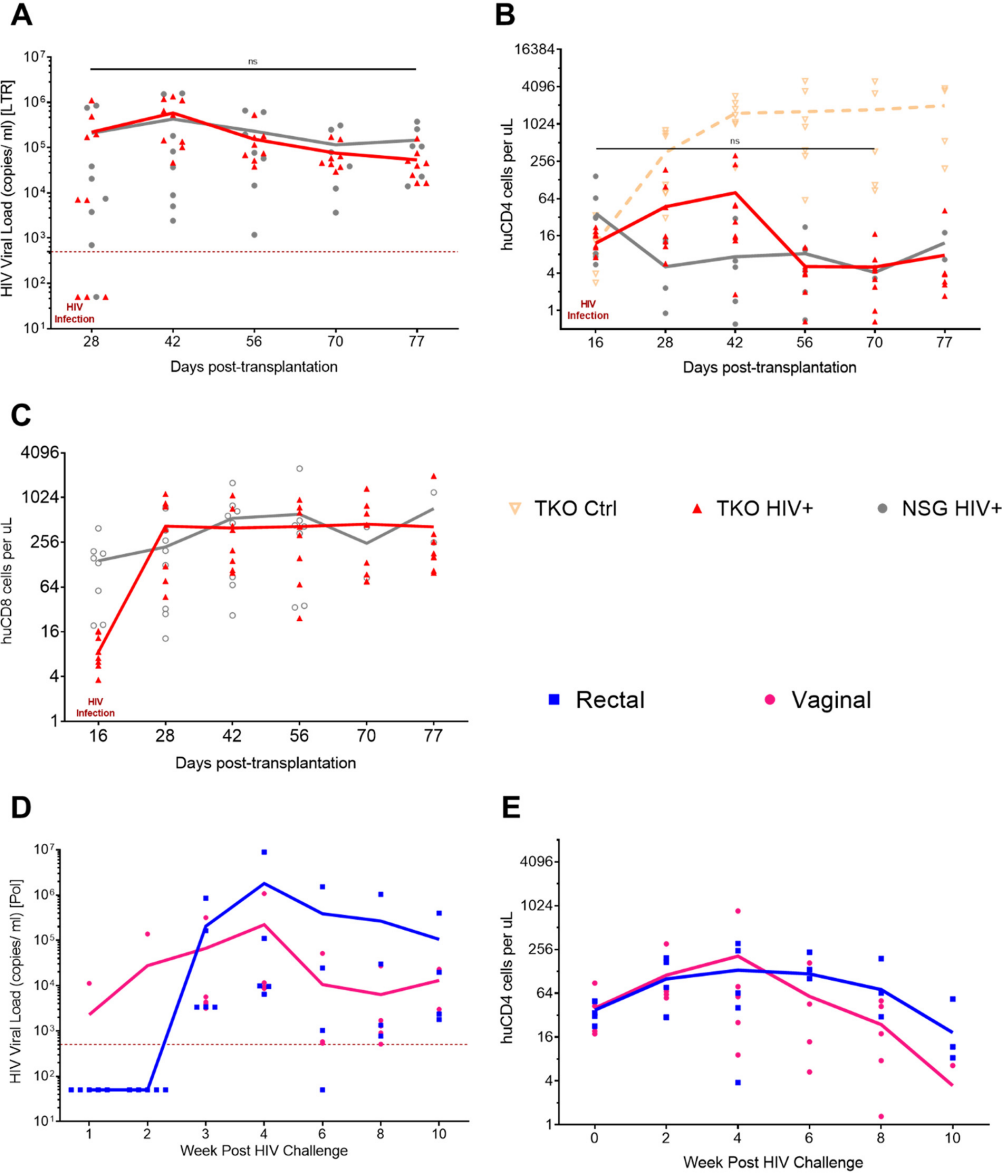
TKO hu-PBMC model supports HIV infection when challenged via i.p., rectal, and vaginal routes. TKO (*n* = 9) and NSG (*n* = 8) mice were transplanted with human PBMC, and 16 days later, mice were challenged i.p. with HIV-1_BaL_ (20 ng p24/mouse). Plasma HIV viremia and CD4^+^ T cells were monitored every 1 to 2 weeks by qRT-PCR and FACS analysis. The TKO hu-PBMC model supports HIV infection with robust plasma viremia (>5 × 10^2^ copies/ml) (A) and loss of CD4^+^ T cells over the 11 weeks (B). CD4^+^ T cell counts from control TKO mice were added to compare HIV infection versus no HIV. The CD8^+^ T cell count remained high throughout infection (C). Viremia in the TKO hu-PBMC model is comparable to the viremia seen in the NSG hu-PBMC model. (Note that mice were lost due to death at days 56 and 70). TKO mice were transplanted with human PBMC and 16 days later were challenged with HIV-1_BaL_ (8 ng p24/mouse). Male mice (*n* = 5) were challenged rectally and female mice (*n* = 5) vaginally. Plasma HIV viremia and CD4^+^ T cells were monitored every 2 weeks by qRT-PCR and FACS analysis. The TKO hu-PBMC model supports HIV infection with robust plasma viremia (>5 × 10^2^ copies*/*mL) (D) and loss of CD4^+^ T cells over 10 weeks (E). (Note that mice were lost due to death at weeks 4 and 8.) Two independent experiments were combined and analyzed. Statistical significance determined by mixed-effects analysis on Prism, with alpha value of 0.05.

To test whether the TKO hu-PBMC model supports HIV-1 infection, we challenged engrafted TKO mice with HIV-1_BaL_. About 2 weeks after transplantation, mice were bled and fluorescence-activated cell sorting (FACS) analysis was done to confirm engraftment of mice. Once confirmed, mice were challenged with 20 ng p24 HIV-1_BaL_ by i.p. injection under general anesthesia. NSG hu-PBMC mice were also challenged in parallel to compare the TKO model with the NSG model. After HIV challenge, plasma viremia and cell composition were monitored every 2 weeks by plasma quantitative PCR (qPCR) and FACS analysis. TKO hu-PBMC mice began to show robust plasma viremia 2 weeks after HIV challenge, and plasma viremia levels were maintained throughout the study with no significant differences from NSG hu-PBMC mice (Fig. 3A). We also monitored CD4^+^ T cell counts to see whether the hu-mouse model reflected depletion of CD4^+^ T cells seen in humans. Starting 2 weeks post-HIV challenge, CD4^+^ T cell counts began to decline in both HIV-infected TKO and NSG mice compared to control TKO mice (Fig. 3B). CD4^+^ T cell counts continued to decline over the remainder of the study. The data show that as plasma viral load increases in the mice, CD4^+^ T cells decrease over time, which accurately models HIV-1 infection in humans. As expected, levels of CD8^+^ T cells remained high during HIV-1 infection, and no significant differences were observed between TKO and NSG models (Fig. 3C).

To test the utility of the model, we challenged mice with HIV-1_BaL_ via rectal and vaginal routes. Similar to the case with intraperitoneal challenge, mice had robust plasma viremia and CD4^+^ T cell depletion when challenged rectally or vaginally with HIV-1 (Fig. 3D and E), although it was delayed compared to intraperitoneal challenge. These results show that TKO hu-PBMC mice challenged intraperitoneally, rectally, or vaginally with HIV-1 are readily infected with HIV-1, leading to robust plasma viremia and depletion of circulating CD4^+^ T cells.

**FIG 4:**
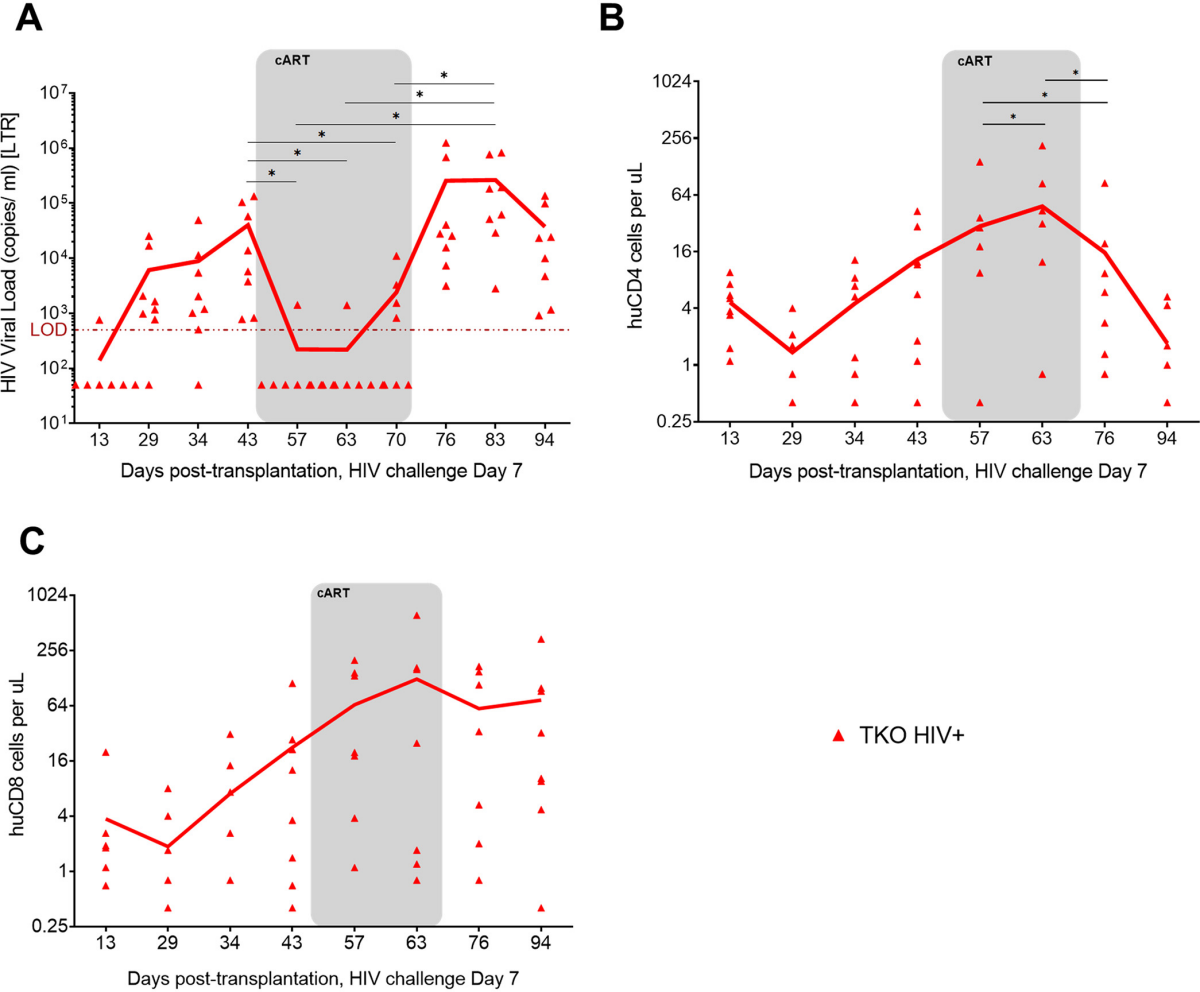
TKO hu-PBMC model response to oral cART with suppression of plasma viremia and CD4^+^ T cell protection. TKO hu-PBMC mice (*n* = 8) were challenged with HIV-1_BaL_ 3 weeks after mice were given oral cART for 4 weeks. Two weeks after the start of oral cART plasma viremia was suppressed while oral cART was given and rebounded once oral cART was interrupted (A). A similar response was seen in the CD4^+^ T cell protection while mice were on oral cART (B). CD8^+^ T cell count remained high pre- and post-oral cART (C). The TKO model response to oral cART is similar to that of the NSG model (historical data). Statistical significance was determined by a one-tailed paired Student’s *t* test, with alpha value of 0.05. *, *P* < 0.05.

Next, we evaluated if HIV-1-infected TKO hu-PBMC mice would respond to oral combinatorial antiretroviral therapy (cART) similarly to humans. In humans, cART medications block the replication of HIV, leading to suppression of viral loads, which, in turn, increases CD4^+^ T cell counts (21). To test the TKO hu-PBMC model, we treated mice orally for 4 weeks with cART composed of drugs that block new infections, without inhibiting viral production in infected cells. The cART regimen consisted of HIV nucleoside analog reverse transcriptase inhibitors (tenofovir disoproxil fumarate [TDF; Truvada] and emtricitabine [FTC]) (Gilead Sciences) and an integrase inhibitor (raltegravir [RAL; Isentress]) (Merck), scaled down to the equivalent mouse dosage using the appropriate conversion factor (22). Medications were powdered, mixed, and suspended in sweetened water solution (Medidrop sucralose; ClearH_2_O) and provided in water bottles to mice as the sole water source. After 2 weeks of oral cART, plasma viral loads were significantly suppressed, with some mice having loads below the limit of detection (Fig. 4A). Plasma viremia was measured weekly while the mice were on oral cART for 4 weeks, and all mice showed suppressed plasma viral loads. With suppressed plasma viremia, CD4^+^ T cell counts increased significantly while the mice received oral cART (Fig. 4B). When oral cART treatment was discontinued, plasma viremia rebounded, followed by a decline in CD4^+^ T cells (Fig. 4A and B). However, levels of CD8^+^ T cells did not significantly change during or after oral cART (Fig. 4C). All of these trends closely model the effects of oral cART in humans. Collectively, these results demonstrate the use of the TKO hu-PBMC model for testing antiviral drugs *in vivo*.

**FIG 5:**
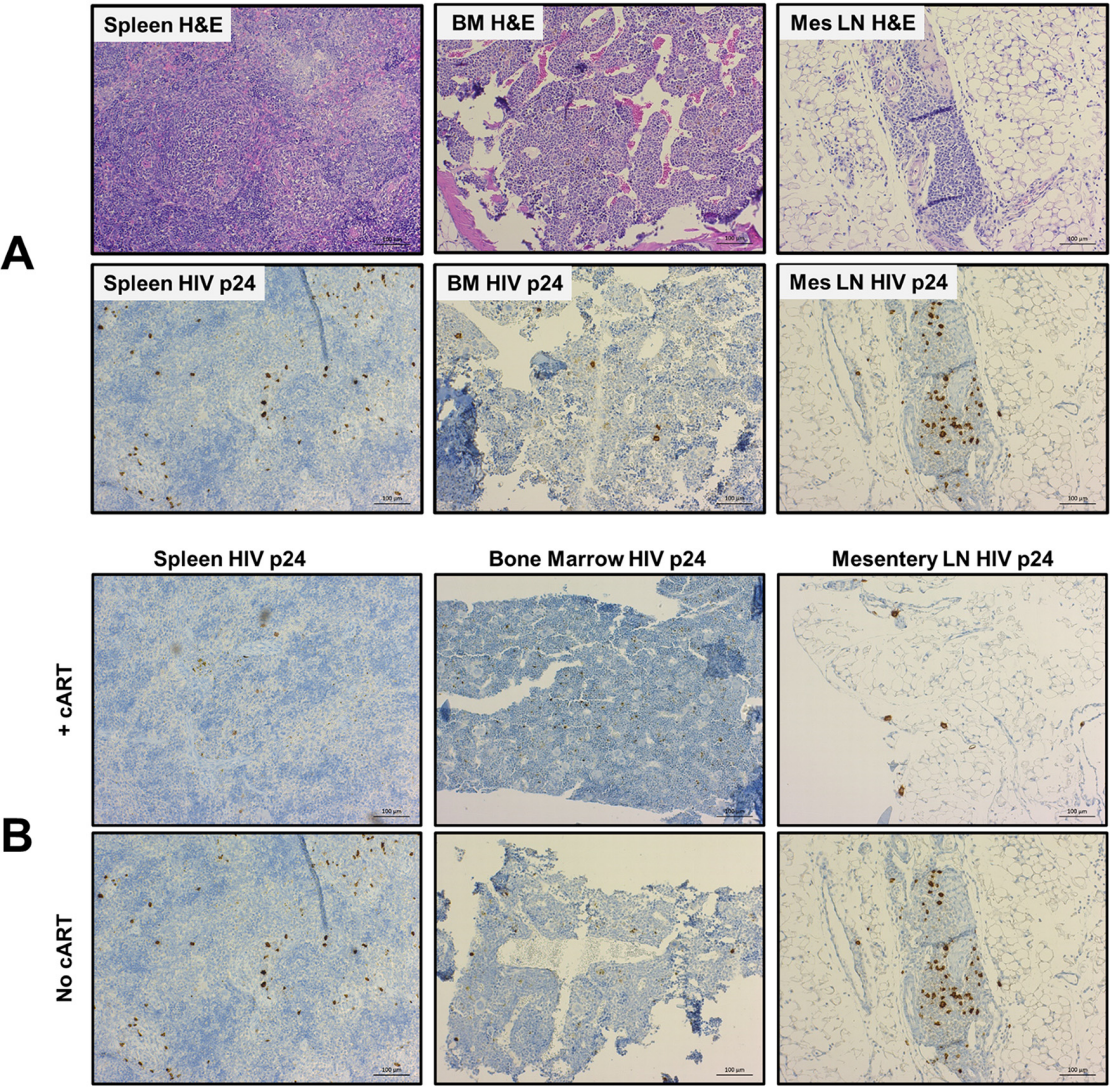
The TKO hu-PBMC model supports HIV infection of lymphoid tissues. TKO mice with robust and consistent peripheral CD45^+^ engraftment and robust plasma viremia were humanely euthanized and tissues (spleen, sternum [bone marrow], and mesenteric lymph node) were collected for histology and IHC. Tissues were processed by the COH Solid Tumor Pathology Core for histology and immunohistochemistry: H&E, huCD3^+^, huCD4^+^, huCD8^+^, and HIV p24. There was positive staining of HIV p24 in lymphoid tissues (A). A cohort of mice was given oral cART for 4 weeks. Mice were euthanized while on oral cART, and IHC analysis was done on lymphoid tissues. Tissues were processed by the COH Solid Tumor Pathology Core for histology and immunohistochemistry: H&E, huCD3^+^, huCD4^+^, huCD8^+^, and HIV p24. There was a qualitative reduction in HIV p24 positive staining for mice receiving oral cART compared to mice not receiving oral cART (B), modeling persistent HIV infection in humans. Representative images from the cohort of mice are shown.

Lastly, we evaluated HIV infection in the lymphoid tissues by immunohistochemistry. At the endpoint of the studies, tissues were collected and submitted for immunohistochemistry (IHC) staining with antibodies against the HIV p24 antigen and human surface markers CD3, CD4, and CD8. There was positive staining for HIV p24 antigen in bone marrow, liver, mesenteric lymph node, spleen, and rectum (Fig. 5A). While the TKO hu-PBMC model supports engraftment of CD4^+^ T cells in the brain (Fig. 2), HIV p24 staining in the brain was inconclusive (data not presented), indicating the need for further studies. Since the persistence of HIV despite ART is a critical factor in HIV cure strategies, we also wanted to evaluate the effect of oral cART on tissue reservoirs of infected mice. During oral cART, a cohort of mice with suppressed plasma viremia was humanely euthanized and tissues were collected for IHC. In this group, there was a qualitative reduction of HIV p24-positive stained cells in the lymphoid tissue reservoirs of the bone marrow, lymph node, and spleen in mice on oral cART relative to untreated mice (Fig. 5B). This indicates that HIV infection persists in the TKO hu-PBMC model despite suppressive oral cART, demonstrating the use of the model to study HIV persistence and treatments.

### The TKO hu-PBMC model has a delayed onset of GVHD compared to the NSG model.

**FIG 6:**
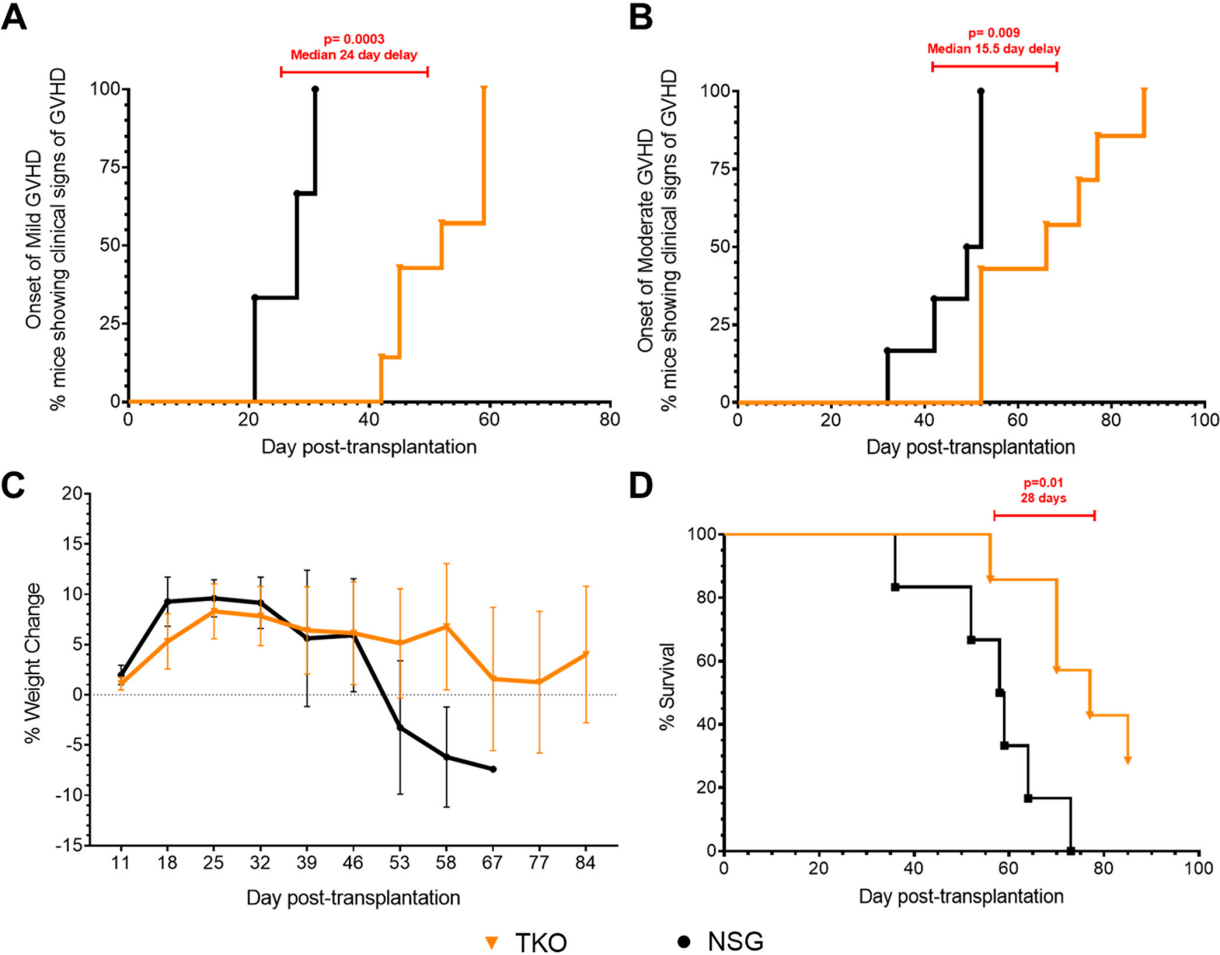
Delayed onset of GVHD in the TKO model compared to the NSG model. TKO (*n* = 7) and NSG (*n* = 6) hu-PBMC mice were monitored twice weekly for clinical signs of GVHD. Mild GVHD was defined as having a slightly ruffled coat with or without slightly hunched posture. Moderate GVHD was defined as having a ruffled coat with or without hunched posture and weight loss of 10 to 20%, with or without alopecia. TKO mice had a 24-day delayed onset of mild GVHD (A) and a 15.5 delay onset of moderate GVHD (B) compared to NSG mice. According to monitoring of weight weekly, TKO mice maintained and lost less weight than NSG mice (C). TKO mice had a 28-day longer survival time than NSG mice (D). Statistical significance was determined by log rank (Mantel-Cox) test on Prism, with alpha value of 0.05.

The major limitation with the NSG hu-PBMC model is the development of graft-versus-host disease (GVHD). To test whether the TKO hu-PBMC model would have a delayed onset of GVHD, clinical signs of GVHD were monitored weekly after transplantation of mice, and the date of the first sign of GVHD was recorded. Based on previous studies (16), we categorized GVHD as mild, moderate, or severe when observing clinical signs. Mild GVHD was defined as having a slightly ruffled coat with or without slightly hunched posture. Moderate GVHD was defined as having a ruffled coat with or without hunched posture and weight loss of 10 to 20%, with or without alopecia. Severe GVHD was defined as having a ruffled coat, hunched posture, and weight loss greater than 20%, with or without alopecia and lethargy. The median onset of mild GVHD clinical signs in NSG mice was 28 days, while in TKO mice it was 52 days (Fig. 6A). The TKO hu-PBMC model has a delayed onset of mild GVHD (24 days) compared to the NSG model. When we monitored for moderate GVHD, the median onset of moderate GVHD clinical signs in NSG mice was found to be 50.5 days, while in TKO mice it was 66 days (Fig. 6B). The TKO hu-PBMC model has a delayed onset of moderate GVHD (15.5 days) compared to the NSG model. Finally, when we monitored for severe GVHD, the TKO hu-PBMC model was found to have a delayed onset (18 days) compared to the NSG model (data not shown). These results show that the TKO hu-PBMC model has a delayed onset of mild, moderate, and severe GVHD compared to the NSG hu-PBMC model.

Weight loss is a clinical sign of GVHD and a major concern for the welfare of the mice (23). We monitored the weight of the mice weekly to measure percent weight loss over time, which revealed that NSG mice had greater and faster weight loss than TKO mice (Fig. 6C). TKO mice were able to maintain their weight over the length of the study, while NSG mice began to lose weight after day 46. Coupled with the weight, we also monitored the survival time of the mice. The date of death was defined as the day mice had to be euthanized for humane endpoints or the day mice were found dead. TKO mice survived an average of 28 days longer than NSG mice over the length of the study (Fig. 6D). All NSG mice were dead by day 73, while two TKO mice survived until the end of the study, day 85. These results show that the TKO hu-PBMC model maintains weight and has a longer survival time than the NSG hu-PBMC model.

**FIG 7:**
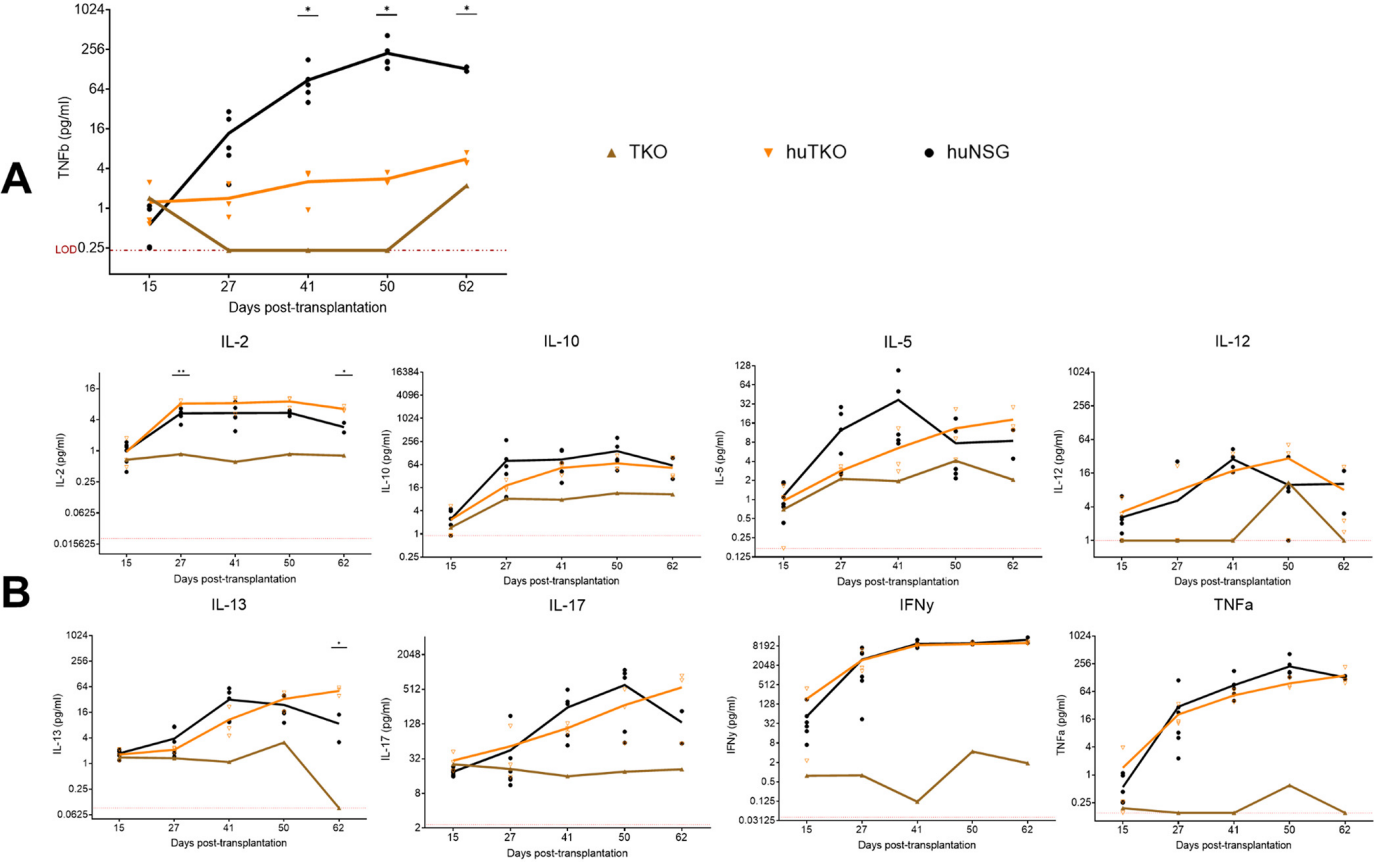
NSG hu-PBMC mice have more circulating plasma TNF-β than TKO hu-PBMC mice. Plasma samples were collected every 2 weeks and submitted to Quansys Biosciences (Logan, UT) to be tested on Quansys Biosciences’ Q-Plex TM human high-sensitivity multiplexed ELISA array. All other human inflammatory cytokines had no difference between TKO (*n* = 3) and NSG hu-PBMC (*n* = 5) mice (B), except for TNF-β. NSG hu-PBMC mice had more circulating plasma TNF-β than TKO hu-PBMC mice (A). Statistical significance was determined by mixed-effects analysis on Prism, with alpha value of 0.05. *, *P* < 0.05.

To evaluate whether there were differences in plasma inflammatory cytokines produced by the TKO and NSG hu-PBMC models, a human inflammatory cytokine assay was performed on the plasma of the mice collected throughout the study. The samples were tested for human interleukin 1α (IL-1α), IL-1β, IL-2, IL-4, IL-5, IL-6, IL-10, IL-12, IL-13, IL-15, IL-17, IL-23, gamma interferon (IFN-γ), tumor necrosis factor alpha (TNF-α), and TNF-β using Quansys Biosciences’ Q-Plex human high-sensitivity multiplexed enzyme-linked immunosorbent assay (ELISA) array. While there were no significant differences in most cytokines assayed (Fig. 7B), the NSG hu-PBMC mice had significantly more plasma TNF-β than the TKO mice (Fig. 7A). The low plasma levels of TNF-β in the TKO hu-PBMC mice may contribute to the delayed onset of GVHD compared to that in the NSG model, as further discussed later.

## DISCUSSION

TKO mice have previously been shown to be graft-versus-host disease (GVHD) resistant when used to develop a hu-BLT mouse model (18); however, that model requires human fetal tissues and has other practical limitations, including its reproducibility and availability to the research community (24). In this study, we developed a novel hu-PBMC mouse model that supports HIV-1 infection and has a delayed onset of GVHD compared to the previously established NSH hu-PBMC model (7).

We observed consistent engraftment of human CD45^+^ leukocytes and CD3^+^ lymphocytes in TKO mice after intraperitoneal transplantation of human PBMC (Fig. 1). Further analysis of T cell subpopulations showed the robust circulation of CD4^+^ and CD8^+^ T cells in the peripheral blood of the TKO hu-PBMC model (Fig. 1). When comparing the model to the commonly used NSG hu-PBMC model, we observed no significant difference in engraftment of human cells between the models. Immunohistochemistry analysis of the tissues showed that the TKO hu-PBMC model supports the engraftment and development of lymphoid tissues, including bone marrow, lymph node, and spleen, with human T cells (Fig. 2 and Table 1). The presence of human T cells in the lymphoid tissues is notable because of the critical role T cells play in the establishment and maintenance of persistent HIV-1 infection in humans.

To test the utility of the novel TKO hu-PBMC model, we challenged mice with HIV-1_BaL_ intraperitoneally, rectally, and vaginally. To our knowledge, this is the first demonstration that a PBMC humanized mouse model reliably supports HIV infection via the rectal and vaginal routes. Infection via all three routes led to robust plasma viremia and depletion of CD4^+^ T cells over time (Fig. 3), thus modeling human pathogenesis. When comparing the TKO and NSG models, we found no significant differences in plasma viremia or CD4^+^ T cell depletion. We also observed robust HIV p24 antibody staining in bone marrow, lymph node, and spleen when we analyzed lymphoid tissue by IHC (Fig. 5A). When we placed the HIV-infected TKO hu-PBMC mice on oral cART for 4 weeks, we observed a suppression of plasma viral viremia and an increase in CD4^+^ T cells (Fig. 4). Once oral cART was stopped, there was a rebound of plasma viremia and CD4^+^ T cell depletion as seen in humans.

Lastly, we observed a delayed onset of GVHD in the TKO hu-PBMC model. CD47, which is known as the “don’t eat me” signal, is regulated by signal regulatory protein α (SIRPα), and the *CD47* knockout induces tolerance for the engrafted human cells by obviating the need for CD47^−^ SIRPα signaling (19). Weekly observation for GVHD clinical signs revealed a median 24-day delay in the onset of mild GVHD and a median 15.5-day delay in the onset of moderate GVHD in the TKO model compared to the NSG model (Fig. 6). We also observed a greater ability of the TKO hu-PBMC mice to maintain their body weights compared to the NSG mice over the length of the study. Survival time was increased in the TKO model compared to that in the NSG model, with TKO hu-PBMC mice having an average 28-day-longer survival time (Fig. 6). We observed significantly lower levels of plasma TNF-β in the TKO hu-PBMC mice than in the NSG mice (Fig. 7). TNF-β, also known as lymphotoxin alpha (LTα), is a TNF superfamily member and plays a crucial role in the development and orchestration of robust immune responses (25). Activated CD4^+^ Th subsets Th1 and Th17, but not Th2, express surface LTα, as do CD8^+^ T cells and B cells (25, 26). LTα has been previously shown to be an important contributor to GVHD pathogenesis (27). Chiang et al. described the use of a humanized anti-LTα monoclonal antibody (MLTA3698A) that specifically depleted activated LT-expressing human donor T and B cells, resulting in prolonged survival times in the hu-SCID mouse model of GVHD (25). Based on these findings, it may be possible that the low production of TNF-β in the TKO hu-PBMC model contributes to the delayed onset of GVHD compared to that in the NSG model. However, further studies are warranted to study the role of TNF-β in the TKO model.

To our knowledge, this is the first demonstration of a TKO hu-PBMC model for HIV-1 infection that can delay the onset of GVHD compared to that in the NSG hu-PBMC model. The delayed onset of GVHD and increased survival time of the TKO hu-PBMC mice make them a valuable tool for studying HIV-1 infection and pathogenesis and evaluating new therapies.

## MATERIALS AND METHODS

### Mice.

Studies were performed with male and female NOD.Cg-*Prkdc^scid^ Il2rg^tm1Wjl^*/SzJ (NSG) mice (JAX stock number 005557) and B6.129S-*Rag2^tm1Fwa^ CD47^tm1Fpl^ Il2rg^tm1Wjl^*/J (TKO) mice (JAX stock number 025730) aged 3 to 5 weeks at the initiation of studies. Mice were group-housed in individually ventilated cages (OptiCages; Animal Care Systems) on corncob bedding (Bed-o’Cobs, 1/8 in.; The Andersons, Maumee, OH) with a square nestlet and polyvinyl chloride (PVC) tube provided for enrichment. Mice were allowed free access to rodent chow (LabDiet 5350) and autoclaved acidified reverse osmosis purified water (pH 2.4 to 2.8) in bottles. After inoculation with HIV, mice were housed under animal biosafety level 2 (ABSL2) conditions, group-housed in static disposable cages (Innocage; Innovive). The room temperature was held at a range of 68 to 79°F, and the room humidity range was 30% to 70%. Mice were designated specific-pathogen free (SPF) for mouse rotavirus, Sendai virus, pneumonia virus of mice, mouse hepatitis virus, minute virus of mice, mice parvovirus, Theiler murine encephalomyelitis virus, mouse reovirus type 3, mouse norovirus, lymphocytic choriomeningitis virus, mouse thymic virus, mouse adenovirus types 1 and 2, mouse cytomegalovirus, polyomavirus, K virus, ectromelia virus, hantavirus, Prospect Hill virus, Filobacterium rodentium, Encephalitozoon cuniculi, Mycoplasma pulmonis, *Helicobacter* spp., and Clostridium piliforme and free of any endo- and ectoparasites. Mice were maintained in accordance with the *Guide for the Care and Use of Laboratory Animals* (28) in a facility accredited by the American Association for the Accreditation of Laboratory Animal Care (AAALAC). All experiments were performed according to the guidelines of the Institutional Animal Committee of the Beckman Research Institute of the City of Hope (COH), IACUC 16095.

### Preparation and engraftment of PBMC.

Human PBMC were obtained from peripheral venous blood of healthy donors from the Apheresis Center at the City of Hope, in accordance with COH institutional review board protocol number 17155. Each animal experiment used a different blood donor (*n* = 5). Human PBMC (1 × 10^7^ cells), purified by density gradient centrifugation using SepMate tubes and Lymphoprep medium (StemCell Technologies, Vancouver, BC, Canada), were resuspended in sterile saline and injected intraperitoneally (i.p.) into mice.

### HIV-1 infection and oral cART therapy.

Once engraftment of mice was confirmed, they were challenged with HIV-1_BaL_ (20 ng p24/mouse) by i.p. injection while under general isoflurane anesthesia. HIV-1_BaL_ cell-free virus was obtained from the NIH HIV Reagent Program (HIV-1_BaL_, ARP-510) and then cultured, the titer was determined, and the virus was frozen. For the rectal and vaginal challenge, mice were anesthetized with isoflurane to reach a deep surgical plane as previously described (29, 30). A pipette was rubbed in the genital area to stimulate the emptying of the rectum. For the rectal challenge, mice were placed in ventral recumbency, and the tail was pulled up to expose the rectum. A 20-μL volume of HIV-1_BaL_ (8 ng p24) was atraumatically pipetted into the rectum. For the vaginal challenge, mice were placed in dorsal recumbency, and the tail was pulled and wrapped around fingers to expose the vagina. A 20-μL volume of HIV-1_BaL_ (8 ng p24) was pipetted atraumatically into the vagina. Mice were held with hindlimbs elevated for 3 to 5 min and then transferred to the home cage to recover in a position with hindlimbs elevated. All procedures involving HIV were performed under biosafety level 2+ (BSL2+) conditions in accordance with protocols approved by the institutional biosafety committee of the City of Hope. Mice were bled by retro-orbital bleeding, and peripheral blood cell populations and plasma viral loads were analyzed periodically using flow cytometry and reverse transcription-quantitative PCR (qRT-PCR).

Infected mice with demonstrable viral infection were treated orally for 4 to 6 weeks with cART composed of drugs that block new infections without inhibiting viral production in infected cells. The cART regimen, consisting of tenofovir disoproxil fumarate (TDF [Truvada]; 300 mg/tablet), emtricitabine (FTC; 200 mg/tablet [Gilead Sciences]), and raltegravir (RAL [Isentress]; 400 mg/tablet [Merck]), scaled down to the equivalent mouse dosage using the appropriate conversion factor, was administered in a drinking water formulation (sweetened water gel, Medidrop sucralose; ClearH_2_O). For 400 mL Medidrop, ½ a TDF tablet and ½ an RAL tablet were crushed to powder and mixed by shaking the bottle to a homogenous solution; medicated water was changed weekly. Doses of cART drugs were calculated based on previous studies using the same delivery system (22). Mice were bled by retro-orbital bleeding, and peripheral blood cell populations and plasma viral loads were analyzed periodically using flow cytometry and qRT-PCR. Mice were bled weekly for qPCR, while FACS analysis was sometimes done every 2 weeks due to limited sample volumes. Mice that had suppressed or undetectable plasma viral loads were followed for 4 to 6 weeks, at which time the cART regimen was withdrawn. The mice were bled and assayed for the rebound of plasma viremia and peripheral cell composition periodically until euthanized.

### Flow cytometry.

Samples of peripheral blood were collected by retro-orbital bleeding under general anesthesia and stained for 30 min with BV711-conjugated antihuman CD3, allophycocyanin (APC)-conjugated anti-human CD4, BB515-conjugated anti-human CD8, and BUV395-conjugated anti-human CD45 (all from BD Biosciences, San Jose, CA). Stained peripheral blood samples were then lysed with red blood cell lysis buffer, and absolute cell counts were calculated using BD liquid counting beads (BD Biosciences). Flow cytometry was performed using a BD Fortessa II instrument (BD Biosciences) and analyzed with FlowJo software.

### Plasma HIV qRT-PCR.

Plasma viremia was assayed using one-step reverse transcriptase real-time PCR (TaqMan assay) with an automated CFX96 Touch real-time PCR detection system (Bio-Rad). We modified the qRT-PCR setup from Sathessan et al. and use their HIV long terminal repeat (LTR) primer set (22). The HIV-1 level in peripheral blood was determined by extracting RNA from blood plasma using the QIAamp viral RNA minikit (Qiagen) and performing TaqMan qPCR using either a primer and probe set targeting the HIV-1 LTR region (FPrimer, GCCTCAATAAAGCTTGCCTTGA; RPrimer, GGCGCCACTGCTAGAGATTTT; probe, 5′FAM/AAGTAGTGTGTGCCCGTCTGTTGTGTGACT/3IABkFQ) or the HIV-1 Pol region (FPrimer, GACTGTAGTCCAGGAATATG; RPrimer, TGTTTCCTGCCCTGTCTC; probe, 5′Cy5/CTTGGTAGCAGTTCATGTAGCCAG/3′IABkFQ], using the TaqMan fast virus 1-step master mix (Applied Biosystems). According to the manufacturer’s instruction (QIAamp viral RNA minikit [Qiagen]), the protocol is designed for purification of viral RNA from minimal 140 μL plasma. In our study, the plasma sample was expanded by dilution (generally 1 to 3 dilutions) because a limited volume of plasma (20 to 60 μL) was available. We calculated our limit of detection (LOD) for both HIV LTR and HIV Pol primer to be ∼500 RNA copies/mL based on our qRT-PCR setup and dilution of samples. Therefore, we defined values below that LOD number as undetectable.

### Immunohistochemistry.

A cohort of mice from each experiment (*n* = 3 to 5) were euthanized for tissue collection. Samples of skin, lung, liver, spleen, small and large intestine, sternum bone marrow, reproductive tract, and brain tissue from mice were examined at the time of euthanasia. Tissues were submitted to the Pathology Solid Tumor Core of Beckman Research Institute at the City of Hope. These samples were fixed with 10% formaldehyde, embedded in paraffin, and stained with hematoxylin and eosin (H&E). H&E slides were analyzed for histological signs of graft-versus-host disease.

Immunohistochemical staining was performed on 5-μm-thick sections from paraformaldehyde (PFA)-fixed, paraffin-embedded tissue using a standard protocol. Primary antibodies used for immunohistochemistry included rabbit anti-human CD3^+^ (SGV6 clone; Ventana), rabbit anti-human CD4^+^ (SP35 clone; Ventana), rabbit anti-human CD8^+^ (SP57 clone; Ventana), mouse anti-human CD20^+^ (L26 clone; Ventana), mouse anti-human CD163^+^ (MRQ-42 clone; Ventana), mouse anti-human CD68^+^ (KP-1 clone; Ventana), mouse anti-human GFAP (GA5 clone; BOND RTU), and mouse anti-HIV p24 (Kal-1 clone; Dako). DAB staining was performed using a Ventana Ultraview DAB (3,3'-diaminobenzidine) detection kit in a Ventana BenchMark XT processor (Ventana Medical Systems). In addition, staining of a nonhumanized mouse with the same primary antibodies was used as an additional control.

### Monitoring of signs of GVHD.

Clinical signs of GVHD were monitored weekly after transplantation of mice, and the date of the first sign of GVHD was recorded. Based on previous studies (16), clinical signs observed and reported were slightly ruffled coat, ruffled coat, slightly hunched posture, hunched posture, lethargy, alopecia, and weight loss ranging from 10 to 20% and greater than 20%. We categorized GVHD as mild, moderate, or severe when observing clinical signs. Mild GVHD was defined as having a slightly ruffled coat with or without slightly hunched posture. Moderate GVHD was defined as having a ruffled coat with or without hunched posture and weight loss of 10 to 20%, with or without alopecia. Severe GVHD was defined as having a ruffled coat, hunched posture, and weight loss greater than 20%, with or without alopecia and lethargy. Lab members with animal husbandry/care experience were in charge of the weekly observations. The onset of mild, moderate, and severe GVHD clinical signs was recorded. When mice presented with severe GVHD clinical signs with extreme lethargy or moribundity, they were humanely euthanized, and the date was recorded. For Meier-Kaplan survival curves, date of death either for humane euthanasia or for when mice were found dead were used.

### Human cytokine multiplexed ELISA.

Plasma samples from mice (40 to 100 μL) were saved and frozen at −80°C at every blood collection time point of the study. Samples were shipped to Quansys Biosciences (Logan, UT) to be tested on Quansys Biosciences’ Q-Plex human high-sensitivity multiplexed ELISA array. The samples were tested for human IL-1α, IL-1β, IL-2, IL-4, IL-5, IL-6, IL-10, IL-12, IL-13, IL-15, IL-17, IL-23, IFN-γ, TNF-α, and TNF-β. Samples were thawed at ambient temperature and then kept cold on ice. Thawed samples were then diluted with the appropriate Quansys sample dilution buffer. Samples were diluted at ratios (sample/total volume) of 1:5 (20%), 1:20 (5%), and 1:100 (1%). Polypropylene low-binding 96-well plates were used to prepare the samples and standards prior to loading the Q-Plex plate. Each dilution was measured in duplicate for a total of 6 wells per sample. Antigen standard curves were performed in duplicate, diluting the antigen standard 1:3 for 10 points with 2 negative points. The sample and antigen standard incubation was extended from 1 h to 2 h, and the detection of secondary antibody incubation was extended from 1 h to 2 h. An image with a 270-s exposure time was captured using a Q-View Imager LS and Q-View software. Levels of luminescent units or pixel intensity units were then measured by the Q-View software. The duplicate standard curves were fit by the Q-View software, which allows for the selection of multiple nonlinear and linear equations to fit the standard curve. Optimal curve fits were determined automatically by the software by evaluating the recovery of the calibrator standards.

### Statistical analysis.

Unless otherwise noted, error bars in all figures represent the standard errors of the means (SEM). GraphPad Prism software was used for statistical analyses. Student’s *t* test, analysis of variance (ANOVA), mixed-effects analysis, or log rank statistical tests were used, and differences were considered statistically significant when the *P* value was <0.05.
